# Potential Mucosal Irritation Discrimination of Surface Disinfectants Employed against SARS-CoV-2 by *Limacus flavus* Slug Mucosal Irritation Assay

**DOI:** 10.3390/biomedicines9040424

**Published:** 2021-04-14

**Authors:** Marco Alfio Cutuli, Antonio Guarnieri, Laura Pietrangelo, Irene Magnifico, Noemi Venditti, Laura Recchia, Katia Mangano, Ferdinando Nicoletti, Roberto Di Marco, Giulio Petronio Petronio

**Affiliations:** 1 Department of Medicine and Health Science “V. Tiberio”, Università degli Studi del Molise, 8600 Campobasso, Italy; m.cutuli@studenti.unimol.it (M.A.C.); a.guarnieri@studenti.unimol.it (A.G.); laura.pietrangelo@unimol.it (L.P.); i.magnifico@studenti.unimol.it (I.M.); n.venditti@studenti.unimol.it (N.V.); laura.recchia@unimol.it (L.R.); giulio.petroniopetronio@unimol.it (G.P.P.); 2 Department of Biomedical and Biotechnological Sciences, University of Catania, 95123 Catania, Italy; kmangano@unict.it (K.M.); ferdinic@unict.it (F.N.)

**Keywords:** animal models, biomarkers, SARS-CoV-2, slug mucosal irritation assay, *Limacus flavus*, yellow pigment, linear discriminant analysis

## Abstract

Preventive measures have proven to be the most effective strategy to counteract the spread of the SARS-CoV-2 virus. Among these, disinfection is strongly suggested by international health organizations’ official guidelines. As a consequence, the increase of disinfectants handling is going to expose people to the risk of eyes, mouth, nose, and mucous membranes accidental irritation. To assess mucosal irritation, previous studies employed the snail *Arion lusitanicus* as the mucosal model in Slug Mucosal Irritation (SMI) assay. The obtained results confirmed snails as a suitable experimental model for their anatomical characteristics superimposable to the human mucosae and the different easily observed readouts. Another terrestrial gastropod, *Limacus flavus*, also known as “ Yellow slug “, due to its larger size and greater longevity, has already been proposed as an SMI assay alternative model. In this study, for the first time, in addition to the standard parameters recorded in the SMI test, the production of yellow pigment in response to irritants, unique to the snail *L. flavus*, was evaluated. Our results showed that this species would be a promising model for mucosal irritation studies. The study conducted testing among all those chemical solutions most commonly recommended against the SARS-CoV-2 virus.

## 1. Introduction

The disinfection practice involves the actuation of physical procedures and/or chemical or biological products to eliminate pathogenic microorganisms. Therefore, it appears the first preventive practice against the development and spreading of infectious diseases caused by various pathogens, among them the SARS-CoV-2 virus [1,2,3].

The SARS-CoV-2 pandemic was immediately declared a global health emergency by the World Health Organization (WHO). A mortality rate between 2 and 2.5%, 122 million cases worldwide, and more than 2.7 million deaths (WHO update 21 March 2021) confirm the infection’s severity [4]. The virus transmission generally occurs from human to human through airborne, Flügge droplets, and contact with contaminated surfaces [5,6]. 

The SARS-CoV-2, being an enveloped virus, is poorly resistant to acids, detergents, disinfectants, drying, and heat, thus it is quite susceptible to disinfection [7,8,9]

Among disinfectants, chemicals are the most widely used, due to the wide availability of products, the cost–benefit ratio, the broad spectrum of action, and the possible employing on many surfaces and objects. In this context, several institutions, both national [10] and international [11,12], have drawn lists of products for surfaces and not on people counteract the coronavirus SARS-CoV-2 when used according to label directions.

The most commonly recommended substances are alcohols, chlorine compounds, hydrogen peroxide, phenols, iodine-based substances, and Quaternary Ammonium Compounds (QACs), each with a different mechanism of action:

- Alcohols, cross-linking, coagulation, and clumping, acting mainly as an aqueous emulsion on membrane proteins;
- Chlorine compounds, oxidation of proteins, carbohydrates, and lipids even at low concentrations;
- Hydrogen peroxide, oxidation of cell membrane components through the formation of peroxide radicals;
- Phenols, cross-linking, coagulating, clumping destroying the cell wall, and blocking of enzymatic activity;
- Iodine-based substances interfere at the level of the respiratory chain by blocking the transport of electrons, thus decreasing the oxygen supply of aerobic microorganisms;
- QACs act as ionic compounds that irreversibly bind membrane phospholipids, altering their permeability [13].

The effectiveness of an antiseptic product should be completed within thirty seconds to one minute. Indeed, the longer is the time required for disinfection to be effective, the greater is the risk that the user will not follow the correct application procedure required.

Since the pandemic earliest moments, the effectiveness of preventive and control measures reported by national and international guidelines in significantly reducing nosocomial infections has been demonstrated [2]. Among these, chemical disinfection played a crucial role in increasing the number of people who frequently handled disinfectants and a higher risk of accidental eyes, mouth, nose, and mucous membranes irritation.

Localized at the interface with the external environment, the mucosa plays a fundamental role as a protective barrier towards microorganisms and substances of a different nature. For this reason, it is not uncommon that oral, nasal, gastrointestinal, vaginal, and rectal mucous membranes can be intentionally or accidentally exposed to xenobiotics that irritate, with microlesions formation, compromising the protective barrier function, especially towards pathogenic microorganisms [14,15,16].

For toxicological evaluation on mucous membranes, vertebrates are mainly used in laboratory routines [17,18]. Although animal experimentation guarantees reliable data, it raises ethical, legal, and scientific high impact implications. For this reason, directives limiting the use of mammals and vertebrates have been issued [19]. Due to these limitations, researchers have begun to use invertebrates for in vivo model studies [20].

To cope with this scenario, a new in vivo assay, “Slug Mucosal Irritation” (SMI), has been proposed, and the snail *Arion lusitanicus* (*A. lusitanicus*) was the first invertebrate used as an alternative to mammals for mucosal toxicology studies [21].

The usage of snails as an alternative model finds its fundamental in mucosal tissue’s anatomy and physiology, which possesses numerous characteristics overlapping with its human counterpart. Moreover, the snail body conformation can be easily analyzed outside instead of inside the organism. From a structural standpoint, the mucosal tissue is non-keratinized with a monolayer outer epithelium composed of non-ciliated cells with microvilli and mucus-secreting glandular cells [22].

In addition to its locomotor, lubricating, and antidehydration functions, mucus is an essential component in external insults or damage protection [23]. These unique properties depend primarily on mucins, molecules that can hydrate and swell up to 100 times in a few fractions of a second [24]. Mucus secretory mechanism depends on chemical or mechanical stimulation of smooth muscle located near the mucus-secreting cells that produce apocrine secretion granules. Upon reaching the extracellular environment, the passage of ions and water allows rapid hydration of mucins [25].

Several comparative test studies Based on *A. lusitanicus* mucus production, showing a high predictive degree of chemicals irritant potential, have been developed.

Among these, the more relevant were focused on nasal discomfort [26], the tolerability of certain excipients on the children’s skin [27], chemicals that cause severe eye damage and irritation [28,29], and also cosmetic formulations biocompatibility [30,31,32,33,34].

Furthermore, the snail model overcomes some of in vitro cell culture assays limitations; due to the lack of a protective barrier formed by mucus, in vitro cell lines are more sensitive to chemicals than the in vivo mucosa. Another significant advantage is assessing tissue damage, which can be evaluated by microscopic examination and the release of several markers, including proteins and enzymes [35,36,37].

The high predictivity of the SMI test was also demonstrated in compared studies with the rabbit mucosal model since overlapping results were observed between the two models [38,39].

*Limacus flavus* (*L. flavus*) (phylum: Mollusca, class: Gastropoda, order: Stylommatophora, family: Limacidae), also known as “Yellow slug” is a pulmonate, hermaphroditic, synanthropic terrestrial gastropod that is autochthonous to the Mediterranean region and widespread worldwide. Colouration varies from yellowish to pinkish-orange, with grey-green spots. It has light blue-grey tubercles, a short crest, and a yellow-white belly (or feet). The mucus produced by the body is yellow, while the belly one is transparent [40,41].

Studies by Cook et al. on the anatomy and histochemistry of the mucus-producing glands of *Limax pseudoflavus*, a snail species closely related to *L. flavus*, demonstrated diffuse yellow granular cells on the dorsal surface. These glands are responsible for the yellow colouration of the dorsal mucus in *L. pseudoflavus* [42]. Moreover, other studies conducted by Chang et al. on the epithelial and mucus-producing cells have shown notable similarities between *L. flavus* and *A. lusitanicus* in mucus production [43].

The use of *L. flavus* in the SMI test has already been reported in some previous studies about polymers biocompatibility [34,44].

Regarding the chemicals’ irritation potential, Dhondt et al. in 2006 compared the use of *L. flavus* versus *A. lusitanicus* by the SMI test to evaluate 28 eye-referring chemicals. In this work, the authors justify this snail’s choice based on several criteria, such as larger size and greater longevity than *A. lusitanicus*. The results showed more outstanding mucus production for *L. flavus* than *A. lusitanicus* [45].

In this study, for the first time, in addition to the standard parameters recorded in the SMI test (i.e., mucus production, weight change, protein content, and LDH production), the secretion of the characteristic yellow mucus pigment of *L. flavus* was evaluated as a readout of the irritation response.

Thus, the study aimed to evaluate whether the characteristics of *L. flavus* could represent reliable readouts for the SMI test. To this end, the assay was implemented and adapted to *L. flavus* features to discriminate the main biocide substances used for surfaces and objects disinfection. The disinfectant solutions most commonly used and recommended for surfaces use only were investigated in this study, among the plethora of chemicals listed in the official guidelines produced by national and international health organizations to counter the spread of SARS CoV-2 [10,11,12].

## 2. Materials and Methods

### 2.1. Chemicals

Phosphate Buffered Saline (PBS) pH 7.4 (cat no. P3813 Sigma-Aldrich), benzalkonium chloride (BAC) 1% (*w*/*v*) (cat no.12060 Sigma-Aldrich, St. Louis, MO, USA), ethanol (EtOH) 70% (*v*/*v*) (cat no. 1.07017 Merck-Supelco, Darmstadt, Germany), isopropanol 95% (*v*/*v*) (cat. no. 34863 Sigma-Aldrich, St. Louis, MO, USA), isopropanol 70% (*v*/*v*) (cat. no. 34863 Sigma-Aldrich, St. Louis, MO, USA), sodium hypochlorite 0.1% (*v*/*v*) (Honeywell Fluka^TM^ cat. no. 71696 Thermo Fisher Scientific, Waltham, MA, USA), hydrogen peroxide 3% (*v*/*v*) (cat. no H1009 Sigma-Aldrich, St. Louis, MO, USA), chlorhexidine 1% (*w*/*v*) (cat. no 282227 Sigma-Aldrich, St. Louis, MO, USA), and iodopovidone 10% (*w*/*v*) (cat. no PVP1 Sigma-Aldrich, St. Louis, MO, USA). All substances were diluted to the final concentration in sterile deionized water.

### 2.2. Collection, Housing, and Identification of Limacus flavus Specimens

Snails were collected in the proximity of Trivento municipality (Campobasso, Molise Italy) and then housed at a temperature between 16 and 18 °C with a photoperiod of 12-12. Each specimen was carefully inspected for macroscopic lesions or damage to the tubercles to exclude ineligible samples.

A preliminary snail identification in the field was carried out by macroscopic evaluation to confirm the body color (green, brown and greyish), the texture of the mantle (a network of yellow spots of ovoid shape), and the tentacles (bluish color). Additionally, both specimens size and position of the respiratory pore (pneumostome) located behind the mantle’s midline were evaluated [46].

### 2.3. SMI Assay

**Figure 1:**
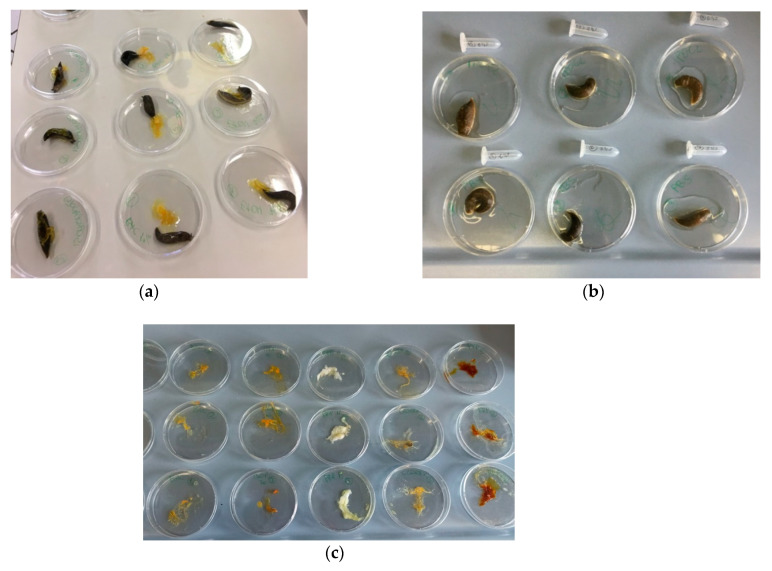
(**a**) Snails during exposure with 100 µL of the test substances; (**b**) snails in contact with PBS after treatment; and (**c**) Petri dishes containing the mucus produced by snails during treatment.

The assay was conducted following Adriaens et al. [28] with some modifications designed to optimize the unique characteristics of the *L. flavus* species. Snails with a weight between 3 and 5 g were selected. Three days before testing, they were housed in a ventilated plastic terrarium lined with PBS (pH 7.4) soaked paper towels and fitted with a wire mesh to drain mucus. Snails before testing were weighed and placed each in a Petri dish. The degree of irritability of the tested biocides was assessed by exposing the snails to 100 µL of each test substance (Figure 1a). 

Unlike the SMI protocol proposed by Adriens et al. [28], test substances were applied to the back and not the snails’ underside. Additionally, as already pointed out by Dhondt et al. [45], given the high mucus production in response to chemicals stimulation, the contact period with the tested substance was reduced from 60 to 15 min. After 15 min, snails were weighed to assess weight loss and transferred to another Petri dish in contact with 1 mL of PBS for 45 min (Figure 1b). Subsequently, samples were collected and frozen at −80 °C for further analysis. 

Treatment-induced weight changes were expressed as percentage weight/weight (% × *w*/*w*) according to the formula:

Bodyweight variation = snail weight after treatment/snail weight before treatment × 100.

Petri dishes in which snails were placed in contact with the test substances were weighed before and after the test to determine the mucus produced by the irritant stimulus (Figure 1c).

The amount of mucus produced after the 15-min contact period (% *w*/*w*) was calculated as follows:

Mucus production = mucus produced after treatment/snail weight before treatment × 100.

Three snails were used for each substance tested, and the experiment was repeated three times independently (three biological and technical replicates).

### 2.4. Mucus Analysis

The mucus produced from each snail after treatment and collected in PBS, henceforth referred to as “sample”, was evaluated for protein quantification, lactate dehydrogenase (LDH) activity, and UV-visible absorbance (λ_max_ 420 nm).

#### 2.4.1. Protein Quantification

Quantification was performed using the Pierce BCA Protein Assay Kit (cat. No. 23227 Thermo-Scientific, Waltham, MA, USA) following the manufacturer’s instructions. Serial dilutions of BSA from 2000 to 20 µg/mL were performed to construct the calibration curve. Of each sample 25 µL were added to 200 µL of the BCA working solution in a microplate. After an incubation period at 37 °C for 30 min, measurements were made with VICTOR3 model1420 Multilabel Counter (PerkinElmer, Waltham, MA, USA) at a wavelength of 562 nm. Results were expressed as µg/mL and normalized for the initial weight of each snail.

#### 2.4.2. LDH

LDH enzyme activity was measured according to Adriaens et al. [35] by the LDH activity assay kit (cat. No. MAK066 Sigma-Aldrich, St. Louis, MO, USA) according to the manufacturer’s instruction. LDH activity is reported as nmol/min/mL = milliunits/mL. One LDH activity unit was defined as the amount of enzyme that catalyzed the conversion of lactate to pyruvate to generate 1.0 mole of NADH per minute at 37 °C.

#### 2.4.3. UV–Visible Spectra

Due to the yellow color of the mucus produced by *L. flavus*, test samples were diluted 1:5 (*V*/*V*) in deionized water and subjected first to spectrophotometric reading from 400 to 600 nm with 1 nm intervals (Lambda25 UV/Vis PerkinElmer); generating UV–VIS spectra for each substance tested.

Curves analysis displayed a maximum absorbance peak for all substances tested at λ_max_ 420 nm, so subsequently, all samples were tested for λ_max_ 420 nm.

### 2.5. Statistical Analysis

Data were expressed as mean ± standard deviation (S.D.) of three biological replicates from three independent experiments. Prism Graph Pad 6 software was used for the one-way ANOVA test, followed by multiple Bonferroni correction tests and linear regression analysis. 

#### Linear Discriminant Analysis

As previously reported by Adriens et al. [17], linear discriminant analysis (LDA) was used as a classification prediction model.

In this study, this PM was applied for discrimination according to mucosal irritation potential of nine surface and object disinfectants prescribed to counteract the spread of SARS-CoV-2. Nine observations (three technical and three biological replicates) were considered for each variable (weight variations, mucus production, protein quantification, LDH activity, and λ_max_ 420 nm to set up the dataset. Two separate LDA models were then developed, one based on the linear combination of the first four variables without λ_max_ 420 nm (LDA w/o λ_max_ 420 nm), the other included the λ_max_ 420 nm spectrophotometric variable (LDA λ_max_ 420 nm). Comparing each model’s confusion matrices (LDA w/o λ_max_ 420 nm and LDA λ_max_ 420 nm) allowed assessment of agreement between observed and predicted categories.

Finally, PBS (control) squared distances (D2) of the LDA λ_max_ 420 nm analysis were used for classification of the tested substances by a numerical score (0–7) and a grade (none to exceptionally high). The significance level was set at *p* = 0.05. XLSTAT software v.2021.1 (Addinsoft Paris, France) was used for LDA analysis.

## 3. Results

### 3.1. SMI Assay

#### 3.1.1. Bodyweight Variation

**Figure 2:**
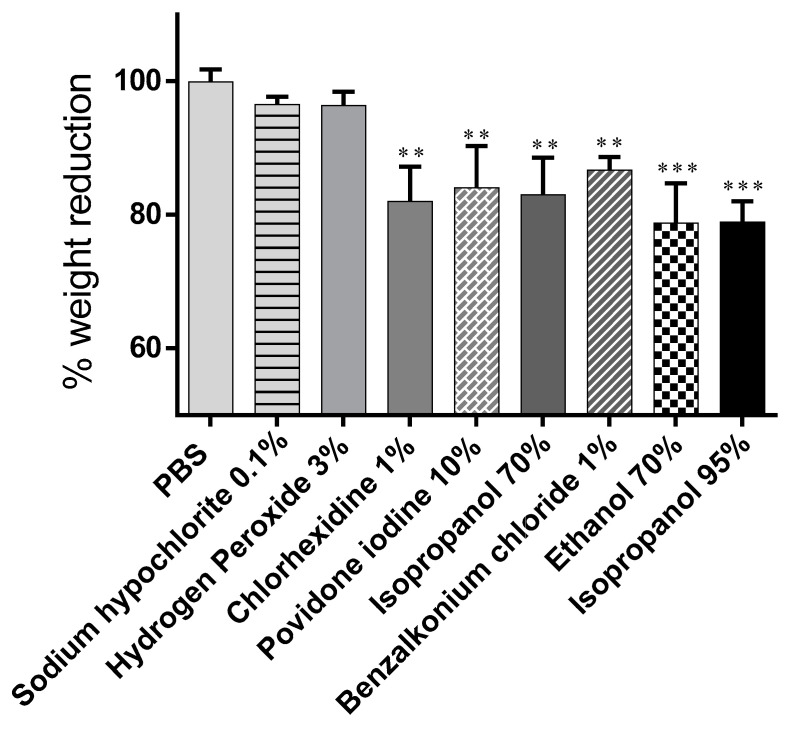
Change in body weight induced by the substances tested. Data are presented as mean ± S.D. and expressed as a percentage of the initial body weight. *** *p* < 0.001, ** *p* < 0.01, significance from control (PBS).

The mean change in weight of snails exposed to the tested disinfectants and controls (PBS pH 7.4, sodium hypochlorite 0.1%, hydrogen peroxide 3%, chlorhexidine 1%, povidone iodine 10%(*w*/*v*), EtOH 70% (*v*/*v*), BAC 1% (*w*/*v*), isopropanol 70%, and isopropanol 95% ranged from 100% to 78.78% (Figure 2).

No change in body weight was observed in snails treated with PBS buffer used as control (100 ± 1.84). In the groups exposed to disinfectants, a weight change was observed caused by the biocidal substances’ irritant action on the snail mucosa (lower percentage values indicate more significant weight loss), among all chemicals tested, EtOH 70% (*v*/*v*) and isopropanol 95% (*v*/*v*) significantly induced higher snail body weight variation by 78.78% ± 5.93% and 78.95% ± 3.10%, respectively. Hydrogen peroxide and sodium hypochlorite had the most negligible influence on body weight change, 96.37% ± 2.05% and 96.53% ± 1.15% (Table 1).

#### 3.1.2. Mucus Production

**Figure 3:**
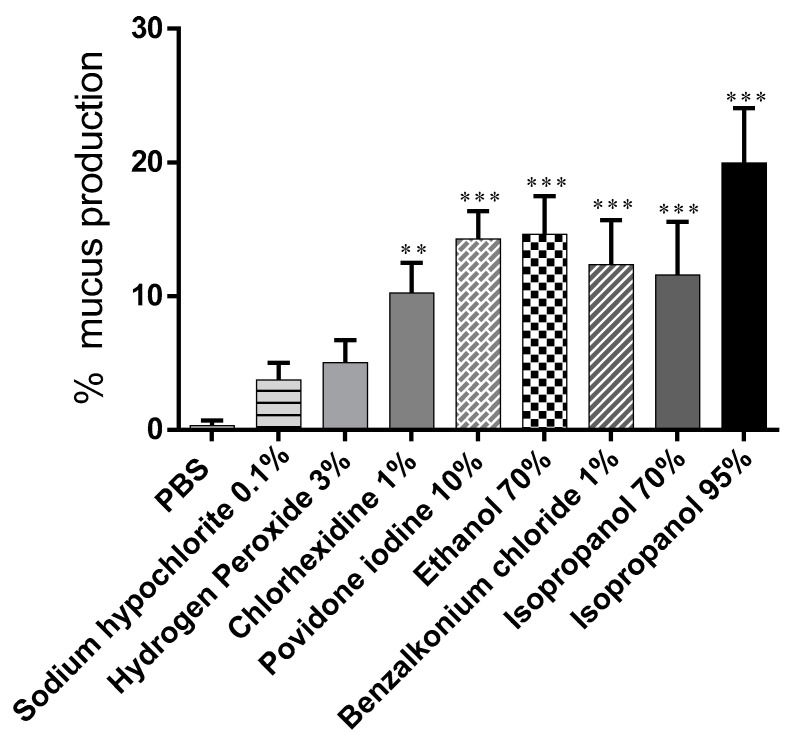
Amount of mucus produced by snails after a 15-min contact period with test substances (percentage relative to initial snail weight). Data are presented as mean ± S.D. *** *p* < 0.001, ** *p* < 0.01 significance from negative control (PBS).

Data on the amount of mucus produced given as a percentage of the snails’ initial weight are shown in Figure 3. Treatment with PBS poorly stimulated mucus production 0.3% ± 0.41%. All substances tested, except hydrogen peroxide (5.03% ± 1.68%) and sodium hypochlorite (3.73% ± 1.27%), induced a significant increase in mucus secretion: EtOH 70% (14.63% ± 2.85%); isopropanol 70% (11.58% ± 3.98%); chlorhexidine 1% (10.23% ± 2.28%); and iodopovidone (14.28% ± 2.07%). Isopropanol 95% was the substance that more stimulated mucus production 19.96% ± 4.08% (Table 1).

#### 3.1.3. Protein Quantification

**Figure 4:**
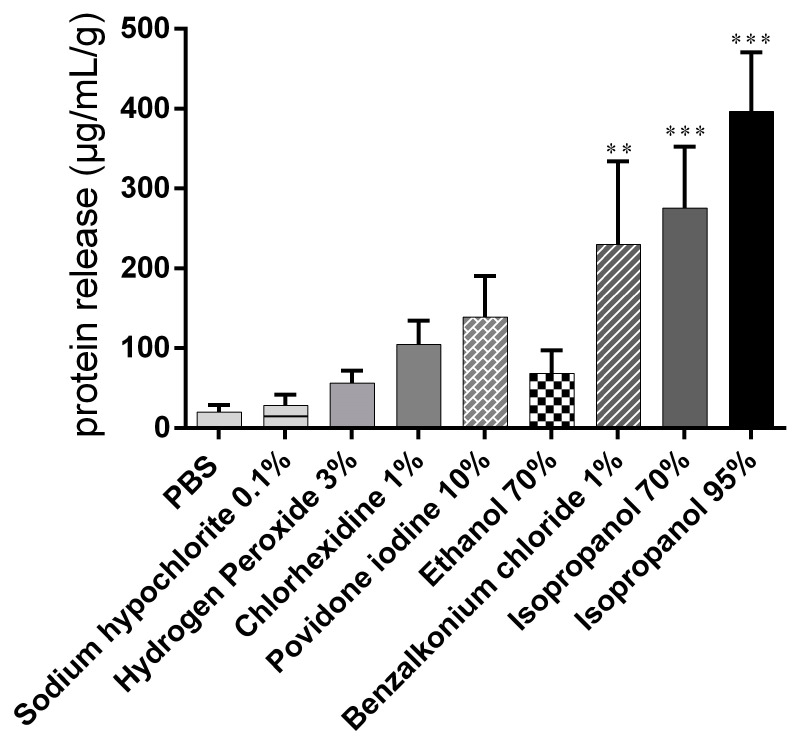
Proteins released from snail mucosa after a 15 min exposure period with the different treatments and subsequent contact with PBS for 45 minutes. Data are presented as mean values ± S.D. and expressed in µg/mL. *** *p* < 0.001, ** *p* < 0.01 significance from negative control (PBS).

Data from protein quantification in samples taken after a 15 min exposure period with the different treatments and subsequent contact with PBS for 1 h were normalized to the snails’ initial weight (Figure 4). 

Moderate protein release into the mucus (20.07 ± 8.68 µg/mL) was recorded in the PBS control group. Treatment with 95% isopropanol, 1% BAC, and 70% isopropanol induced significant protein release compared with the control. The lowest protein concentrations among the biocidal substances tested were determined in the samples from the groups exposed to hydrogen peroxide and hypochlorite (Table 1).

#### 3.1.4. LDH

The LDH activity in mucus correlates with cellular damage caused by snail exposure to test substances.

**Figure 5:**
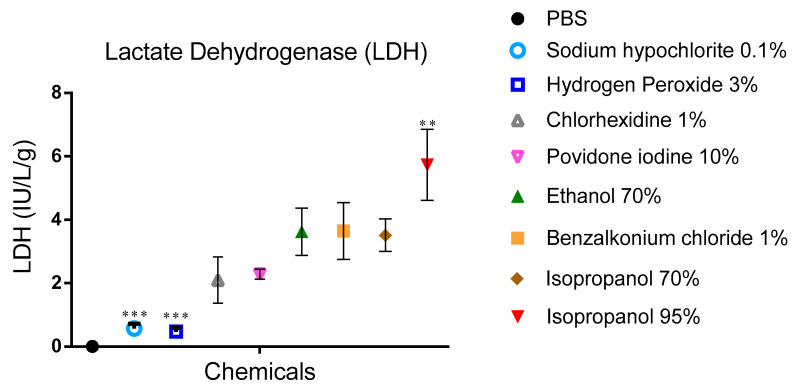
LDH activity in PBS samples after a period of contact with the different biocides for 15 min. Data are presented as mean values and expressed in units/mL PBS per gram body weight. Two-way ANOVA comparing tested substances versus BAC 1% (mean LDH 3.6 IU/LG/g) as already reported by Adriaens et al. [17]. *** *p* < 0.001, ** *p* < 0.01 significance from control (BAC 1%).

For PBS-treated snails, no LDH activity was recorded (Figure 5). On the other hand, 95% isopropanol exposure showed significant activity (5.73 ± 1.13 IU/L.G./g), hydrogen peroxide, and sodium hypochlorite induced less LDH activity 0.47 ± 0.12 and 0.57 ± 0.15IU/L.G./g, respectively.

#### 3.1.5. UV–VIS Spectra

Table 1 shows the λ_max_ 420 nm values. Lower absorbance values were found for the PBS (0.028 0.006), sodium hypochlorite 0.1% (0.035 ± 0.005), and hydrogen peroxide 3% (0.048±0.006) groups while the highest values were found for the isopropanol 95% (0.394 ± 0.019) isopropanol 70% (0.330 ± 0.020) and BAC 1% (0.0301 ± 0.015) groups. 

**Figure 6:**
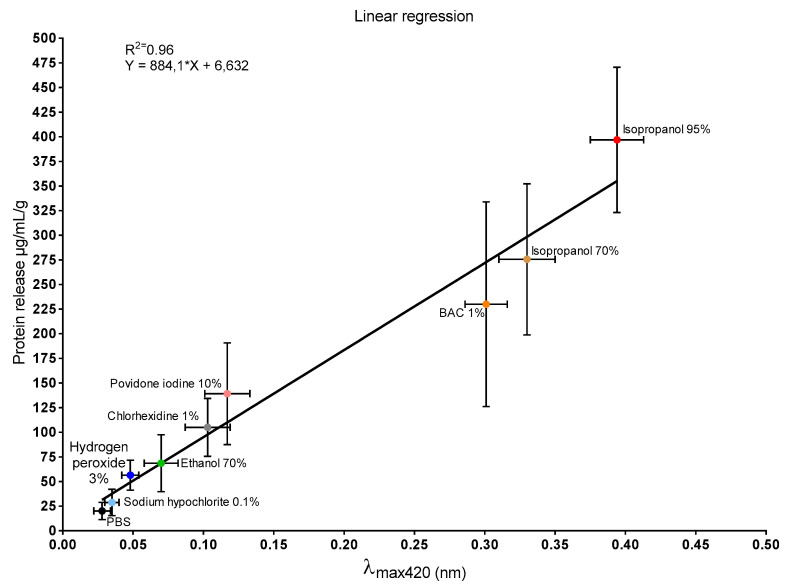
Linear regression analysis between λ_max_ 420 nm values and mucus protein concentration (R^2^ = 0.96; Y = 884.1 * X + 6.632). Data are expressed as mean ± standard deviation (vertical bars protein concentration, horizontal bars λ_max_ 420 nm).

Additionally, a linear correlation between λ_max_ 420 nm and mucus protein was observed (Figure 6).

### 3.2. Linear Discriminant Analysis

**Figure 7:**
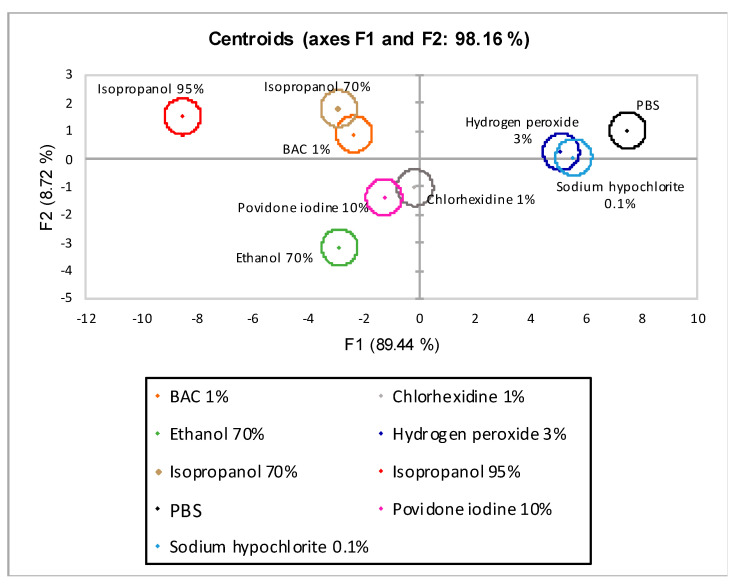
Centroids graph for linear discriminant analysis (LDA) w/o λ_max_ 420 nm. F1 (*x*-axis) and F2 (*y*-axis) are the factor axes extracted from the original variables. For each factor, the percentage of discrimination, both individual and cumulative, is reported in brackets.

The centroid plot of LDA without λ_max_ 420 nm is shown in Figure 7. On the axes were the variables linear combinations with the highest discriminated percentage extracted from the analysis. On the *x*-axis was factor one (F1) with 89.44% discrimination and on the *y*-axis was factor two (F2) with 8.72%. The sum of the two factors (F1 and F2) reached a total bias of 98.16%.

Of the nine disinfectants tested, three were discriminated entirely (PBS, EtOH 70%, and isopropanol 95%), while the remaining were grouped into three distinct groups (hydrogen peroxide 3%-sodium hypochlorite 0.1, chlorhexidine 1%, povidone-iodine 10%, and BAC 1%-isopropanol 70%).

**Figure 8:**
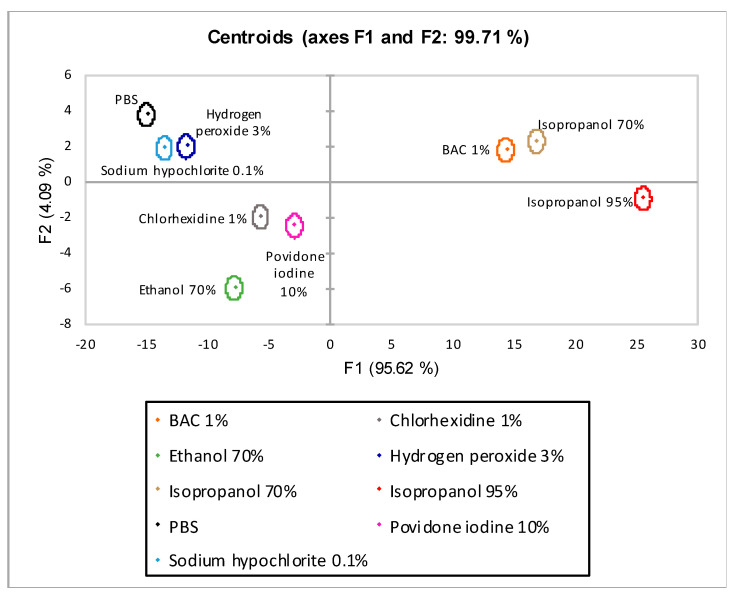
Centroids graph for LDA λ_max_ 420 nm. F1 (*x*-axis) and F2 (*y*-axis) are the factor axes extracted from the original variables. For each factor, the percentage of discrimination, both individual and cumulative, is reported in brackets.

The centroid plot of the LDA with λ_max_ 420 nm is shown in Figure 8. The axes are shown the linear combinations of the variables under investigation with the highest discriminated percentage extracted from the analysis. On the *x*-axis is factor one (F1) with 95.62% discrimination and on the *y*-axis is factor two (F2) with 4.09%. The sum of the two factors (F1 and F2) achieved total percentage discrimination of 99.71%. All nine disinfectants tested were completely discriminated. 

Table 2 shows the percentage of correct predictions broken down for each class (disinfectants tested) for the two models analyzed (λ_max_ 420 and w/o λ_max_ 420) together with the percentage differences.

The introduction of the. Variable λ_max_ 420 resulted in a percentage increase in total correct predictions of 16.05%. The classes that showed the most significant increase in prediction accuracy were BAC 1% (55.55% increase), povidone-iodine 10% (33.33%), and isopropanol 70% (22.22%). Sodium hypochlorite 0.1%, hydrogen peroxide 3%, and EtOH 70% had a modest increase (11.11%), while PBS, chlorhexidine 1%, and isopropanol 95% had no increase.

## 4. Discussion

The main SARS-CoV-2 routes of transmission are interhuman airborne and contact with contaminated surfaces. Thus, healthcare and community prevention measures include regular use of surface disinfectants and hand sanitizers [47,48,49].

The persistence of coronavirus on surfaces has caused the intensive use of chemical disinfectants by healthcare professions and the general public [50,51].

According to Van Doremalen et al. [52], SARS-CoV-2 could remain viable in aerosols for three hours with a low infectious load reduction. The viable virus was shown to be more stable on plastic and stainless steel and could be detected up to 72 h after deposition, although a substantial decrease in viral load was observed. A rough estimate of the median half-life was 5.6 h on steel and 6.8 h on plastic. No viable virus was detected after 4 h on copper or after 24 h on cardboard.

Due to the vast resonance of the pandemic and the high number of confirmed cases worldwide [4], people easily take inappropriate actions caused by fear. Numerous studies have reported improper use of chemical surface disinfectants prescribed against the spread of SARS-CoV-2 by not properly following the manufacturers’ instructions [53], with an increased poisoning risk rate during the year 2020 [54].

Besides, a prepandemic study conducted by Casey et al. [55] had already established a correlation between routine use of chemical disinfectants and mucosal irritant effects together with respiratory health. This study had concluded that the risks of mucosal irritation and asthma are higher in healthcare workers. Thus, repeated exposure to disinfectants had to be evaluated as an occupational disease risk factor when drafting healthcare disinfection protocols [56].

In this scenario, the study aimed at the discrimination according to mucosal irritation potential of nine disinfectants used for objects and surfaces against the spread of SARS-CoV-2 [10,11,12].

To this end, the land snail *L. flavus* was evaluated as a test organism in place of *A. lusitanicus*. In addition to the characteristics common to *A. lusitanicus* (i.e., mucosal epithelium, easily observable irritant effect, vulnerability to mechanical, or chemical damage), this species possesses unique features that promote its adoption as an “ideal” candidate for the evaluation of irritant potential [43,44,45].

As in the *A. lusitanicus* model, mucus production, weight changes, protein content, and LDH activity were evaluated as the main readouts [57].

For the first time in this study, mucus staining was used as a readout to assess mucosal irritant potential. Although the pigment’s chemical nature is unknown, this phenomenon has already been described in other land snail species. Some snails of the genus *Ariolimax* have bright yellow colouration [58]. *Arion fasciatus* (Nilsson) also possesses yellow-orange mantle pigments lost when reared on carrots, lettuce, or paper [59]. Moreover, besides the studies conducted by Cook et al. [42] on *L. pseudoflavus*, yellow pigmentations were evaluated by Seki et al. for the taxonomic distinction of two sibling snails species *Bradybaena pellucida* and *B. similaris* [60].

UV–Vis spectra of mucus samples retrieved from the disinfectant-treated snail (data not shown) revealed a maximum absorbance peak at 420 nm (λ_max_). These absorbance values were correlated with the respective data obtained from mucus protein quantification (Figure 6). Linear correlation analysis demonstrated a direct proportionality between the two variables (R^2^ = 0.96). These findings support the hypothesis that, similar to proteins [61], the pigment is released under stressful conditions.

Evaluation of mucosal irritant potential based on the comparison of individual readouts provided a difficult results interpretation (Table 1). However, a correlation between yellow pigmentation and protein release was demonstrated (Figure 6, the same was not true for % weight variations (Figure 2), % mucus production (Figure 3), and LDH activity (Figure 5).

For this reason, the mucosal irritation potential discrimination of disinfectants (classes) under investigation was assessed by a classification prediction model (LDA) that accounted for both the individual readouts (observations) and a statistically significant combination of them *p* < 0.05.

Two different LDA analyses were performed; in the first one (LDA w/o λ_max_ 420), four observations common to both snail species (% weight variations, % mucus production, LDH, and protein quantification) were taken into account. This model’s total discrimination rate, defined as the sum of observations linear combinations (axes F1 and F2), was 98.16%. Nevertheless, only three of the nine observations were fully discriminated (Figure 7).

In the second model (LDA λ_max_ 420), yellow mucus pigmentation (λ_max_ 420) unique to *L. flavus* was included. The introduction of absorbance data into the model has increased both its discriminatory power (axes F1 and F2 99.71%) and predictive ability. Indeed, all nine observations were discriminated (Figure 8). 

The predictive ability of both models has been reported in Table 2. The correct predictions (% correct) are summarized in percentages and divided both for each class (disinfectants tested) and total prediction. Comparison of the processed models (∆% correct) showed an increase in predictive ability for both total classes (16.05%) and six of the nine chemicals tested. 

These findings demonstrate the contribution of the data obtained from the spectrophotometric analysis of *L. flavus* pigment in class discrimination according to their mucosal irritation potential.

Due to the better discrimination and predictive ability of the LDA λ_max_ 420 model, its D^2^ data (Mahalanobis distance) from PBS (D^2^ PBS LDA λ_max_ 420) were used to classify the tested disinfectants according to mucosal irritation potential (Table 3). 

This parameter was chosen for its statistical meaning. When the D^2^ value between an observation and the group’s centre (calculated as mean) was the smallest, that observation could be classified into that group. The LDA provided D^2^ values for each group, called the linear discriminant function. For each observation, the group with the smallest D^2^ had the largest linear discriminant function, and the observation was classified into that group [62].

Since PBS was used as a control (no irritation potential), its Mahalanobis distance values were selected for classification.

By the D^2^ range values, a classification among the tested disinfectants was set up (Table 3) according to a numerical score (from 0 to 7) and an effects grade (from none to exceptionally high).

According to Dhont et al., *L. flavus* could be used to assess the irritant potential of chemicals without affecting the *A. lusitanicus* SMI concordance and specificity [45].

Nevertheless, in the present study, several chemical disinfectants at different concentrations prescribed by international disinfection protocols have been tested [10,11,12].

For this reason, the *L. flavus* SMI protocol was optimized to introduce a new readout (λ_max_ 420).

Among the substances tested, only EtOH, chlorhexidine, and isopropanol have been previously analyzed by SMI studies (except PBS and BAC employed as control substances) [17,28,45,57,63]. For these substances, given the different concentrations used, a direct comparison of mucosa irritant potentials was not possible.

Nevertheless, although the concentration of isopropanol tested in previous studies was much lower than the current one (10% vs. 70 and 95%), it was already tagged as an irritant confirming snail mucosa high toxicity [28].

Regarding BAC 1%, both the classification proposed in this work (*L. flavus* SMI) and that proposed by previous works (*A. lusitanicus* SMI) were in agreement (very irritating to mucous membranes) [17].

## 5. Conclusions

In conclusion, our results confirmed that this native Mediterranean species might be a viable alternative in the SMI assay [43,44,45]. Furthermore, for the first time in this study, *L. flavus* mucus “yellow pigment” was evaluated as a new readout. Although further studies on the chemical composition and release patterns of this pigment are needed to elucidate the mechanisms of response to chemical insults, it demonstrated a strong correlation with protein secretion under stress conditions and a remarkable capacity in discriminating the mucosal irritant potential of nine surface disinfectants used to counteract the spread of SARS-CoV-2.

## Figures and Tables

**Table 1:** SMI test results for all disinfectants tested. Data are expressed as mean ± standard deviation (S.D.), each value with its unit of measure; ^a,b^ protein quantification and LDH were normalized to the weight of the snails before treatment.

Chemicals	Bodyweight Variation (*w*/*w*%)	Mucus Production (*w*/*w*%)	Protein Quantification (µg/mL)/g ^a^	LDH (iu/l)/g ^b^	λ_max_ 420 (nm)
**PBS**	100.00 ± 1.84	0.30 ± 0.41	20.07 ± 8.68	-	0.028 ± 0.006
**Sodium Hypochlorite 0.1%**	96.53 ± 1.15	3.73 ± 1.27	28.72 ± 13.36	0.57 ± 0.15	0.035 ± 0.005
**Hydrogen Peroxide 3%**	96.37 ± 2.05	5.03 ± 1.68	56.47 ± 15.21	0.47 ± 0.12	0.048 ± 0.006
**Chlorhexidine 1%**	82.04 ± 5.19	10.23 ± 2.28	104.90 ± 29.35	2.10 ± 0.73	0.103 ± 0.016
**Povidone Iodine 10%**	84.09 ± 6.22	14.29 ± 2.07	139.11 ± 51.58	2.28 ± 0.16	0.117 ± 0.016
**EtOH 70%**	78.78 ± 5.93	14.63 ± 2.85	68.64 ± 28.85	3.62 ± 0.75	0.070 ± 0.012
**BAC 1%**	86.68 ± 1.98	12.36 ± 3.32	230.02 ± 103.95	3.64 ± 0.90	0.301 ± 0.015
**Isopropanol 70%**	83.04 ± 5.54	11.58 ± 3.98	275.53 ± 76.64	3.51 ± 0.51	0.330 ± 0.020
**Isopropanol 95%**	78.95 ± 3.10	19.96 ± 4.08	396.87 ± 73.77	5.73 ± 1.13	0.394 ± 0.019

**Table 2:** Training sample percentage of correct predictions for the LDA analysis performed. LDA λ_max_ 420: analysis with λ_max_ 420 variable; LDA w/o λ_max_ 420: analysis without λ_max_ 420; % correct LDA λ_max_ 420—% correct LDA w/o λ_max_ 420: the difference between the two analysis.

Chemicals	% Correct LDA λ_max_ 420	% Correct LDA w/o λ_max_ 420	∆% Correct (% Correct LDA λ_max_ 420– % Correct LDA w/o λ_max_ 420)
**PBS**	100	100	-
**Sodium Hypochlorite 0.1%**	88.89	77.78	11.11
**Hydrogen Peroxide 3%**	77.77	66.67	11.11
**Chlorhexidine 1%**	88.89	88.89	-
**Povidone Iodine 10%**	100	66.67	33.33
**EtOH 70%**	100	88.89	11.11
**BAC 1%**	77.78	22.22	55.55
**Isopropanol 70%**	100	77.78	22.22
**Isopropanol 95%**	100	100	-
**Total**	**92.59%**	**76.54%**	**16.05**

**Table 3:** Disinfectant discrimination according to the mucosal irritation potential.

Chemicals	D^2^ PBS LDA λ_max_ 420	Range	Score	Grade
**PBS**	5	0 to 5	0	none
**Sodium Hypochlorite 0.1%**	11	11 to 20	1	low
**Hydrogen Peroxide 3%**	20			
**Chlorhexidine 1%**	132	20 to 132	2	mild
**Povidone Iodine 10%**	155	132 to 155	3	average
**EtOH 70%**	196	155 to 196	4	average-high
**BAC 1%**	878	196 to 878	5	high
**Isopropanol 70%**	1031	878 to 1031	6	very high
**Isopropanol 95%**	1676	1031 to 1676	**7**	extremely high

## Data Availability

Not applicable.
